# CARE PARTNERS OF PERSONS WITH DEMENTIA USE OF SHARED ACCESS TO THE PATIENT PORTAL

**DOI:** 10.1093/geroni/igad104.0575

**Published:** 2023-12-21

**Authors:** Kelly Gleason

**Affiliations:** Johns Hopkins, Baltimore, Maryland, United States

## Abstract

The patient portal plays a significant and growing role in health system navigation, and most patient portals offer shared access for care partners. Care partners are at the forefront of dementia care, yet little is known about their use of the portal through shared access. We conducted an observational cohort study of patient portal utilization for a 5-year period (10/3/2017-10/2/2022) at an academic health system. The cohort consisted of 49,382 patients ages 65+ with 2+ visits in 24 months. More than half (57.34%) of the cohort was female and white (67.19%) and the average age was 76.56 (SD 8.60). We operationalized diagnosed dementia from ICD10 codes and problem lists. Shared access use was defined as activity happening through “proxy” credentials. We operationalized the patient portal activity metric as the ratio of portal sessions in relation to number of clinical encounters. Older adults with (versus without) diagnosed dementia were similarly likely to be registered for the patient portal (71.20% versus 71.51%, p=0.16) but more likely to have a shared access user (10.41% vs. 3.31%; p<0.001). Among patients with dementia, those who had a shared access user, compared to those who did not, had 341.32 versus 164.33 patient portal sessions, sent 47.56 versus 27.42 messages, and had a 19.22 versus 11.22 patient portal activity metric over the 5-year period (p<0.001 all contrasts). Policy initiatives and organizational efforts that encourage use of shared access may benefit care quality of persons with dementia and their care partners.

